# Author Correction: Global phylogenetic analysis of *Escherichia coli* and plasmids carrying the *mcr-1* gene indicates bacterial diversity but plasmid restriction

**DOI:** 10.1038/s41598-020-58953-0

**Published:** 2020-02-14

**Authors:** Sébastien Matamoros, Jarne M. van Hattem, Maris S. Arcilla, Niels Willemse, Damian C. Melles, John Penders, Trung Nguyen Vinh, Ngo Thi Hoa, Martin C. J. Bootsma, Perry J. van Genderen, Abraham Goorhuis, Martin Grobusch, Nicky Molhoek, Astrid M. L. Oude Lashof, Ellen E. Stobberingh, Henri A. Verbrugh, Menno D. de Jong, Constance Schultsz

**Affiliations:** 1 Department of Medical Microbiology, Academic Medical Center (AMC), Amsterdam, The Netherlands; 2 Department of Medical Microbiology and Infectious Diseases, Erasmus University Medical Center, Rotterdam, The Netherlands; 3 School for Public Health and Primary Care (Caphri), Department of Medical Microbiology, Maastricht University Medical Center (MUMC), Maastricht, The Netherlands; 4 School for Nutrition and Translational Research in Metabolism (NUTRIM), MUMC, Maastricht, The Netherlands; 5 Department of Global Health-Amsterdam Institute for Global Health and Development, AMC, Amsterdam, The Netherlands; 6 Oxford University Clinical Research Unit, Centre for Tropical Medicine, Ho Chi Minh City, Vietnam; 7 Centre for Tropical Medicine, Nuffield Department of Medicine, University of Oxford, Oxford, UK; 8 Julius Centre for Health Sciences and Primary Care, University Medical Centre Utrecht, Utrecht, The Netherlands; 9 Department of Mathematics, Faculty of Science, Utrecht University, Utrecht, The Netherlands; 10 Department of Internal Medicine, Havenziekenhuis - Institute for Tropical Diseases, Rotterdam, The Netherlands; 11 Center of Tropical Medicine and Travel Medicine, Academic Medical Centre (AMC), Amsterdam, The Netherlands

Correction to: *Scientific Reports* 10.1038/s41598-017-15539-7, published online 10 November 2017

In the original version of this Article, Martin C. J. Bootsma, Perry J. van Genderen, Abraham Goorhuis, Martin Grobusch, Nicky Molhoek, Astrid M. L. Oude Lashof, Ellen E. Stobberingh & Henri A. Verbrugh were incorrectly listed as the COMBAT consortium.

This error has now been corrected in the HTML and PDF versions of the Article, and in the accompanying supplementary material.

