# Supplementary material for: Global phylogenetic analysis of Escherichia coli and plasmids carrying the mcr-1 gene indicates bacterial diversity but plasmid restriction
Source: Sci Rep. 2017 Nov 10;7:15364. doi: 10.1038/s41598-017-15539-7 (PMC5681592; doi:10.1038/s41598-017-15539-7)
Supplement: Supplementary file 1 — Supplementary Information [file 41598_2017_15539_MOESM1_ESM.pdf]

Global phylogenetic analysis of *Escherichia coli* and plasmids carrying the *mcr-1* gene indicates bacterial diversity but plasmid restriction

Supplementary Methods and Results

Sébastien Matamoros[1], Jarne M. van Hattem[1], Maris S. Arcilla[2], Niels Willemse[1], Damian C. Melles[2], John Penders[3,4], Trung Nguyen Vinh[1,5,6], Ngo Thi Hoa[6,7], Martin C. J. Bootsma[8,9], Perry J. van Genderen[10], Abraham Goorhuis[11], Martin Grobusch[11], Nicky Molhoek[10], Astrid M. L. Oude Lashof[3], Ellen E. Stobberingh[3], Henri A. Verbrugh[2], Menno D. de Jong[1] & Constance Schultsz[1,5]

1. Department of Medical Microbiology, Academic Medical Center (AMC), Amsterdam, The Netherlands.
2. Department of Medical Microbiology and Infectious Diseases, Erasmus University Medical Center, Rotterdam, The Netherlands.
3. School for Public Health and Primary Care (Caphri), Department of Medical Microbiology, Maastricht University Medical Center (MUMC), Maastricht, The Netherlands.
4. School for Nutrition and Translational Research in Metabolism (NUTRIM), MUMC, Maastricht, The Netherlands.
5. Department of Global Health-Amsterdam Institute for Global Health and Development, AMC, Amsterdam, The Netherlands.
6. Oxford University Clinical Research Unit, Centre for Tropical Medicine, Ho Chi Minh City, Vietnam.
7. Centre for Tropical Medicine, Nuffield Department of Medicine, University of Oxford, Oxford, UK.

8. Julius Centre for Health Sciences and Primary Care, University Medical Centre Utrecht, Utrecht, The Netherlands.
9. Department of Mathematics, Faculty of Science, Utrecht University, Utrecht, The Netherlands.
10. Department of Internal Medicine, Havenziekenhuis - Institute for Tropical Diseases, Rotterdam, The Netherlands.
11. Center of Tropical Medicine and Travel Medicine, Academic Medical Centre (AMC), Amsterdam, The Netherlands.

*S.M. and J.M.v.H. contributed equally to this work.

*Adress correspondence to Sébastien Matamoros (s.p.matamoros@amc.nl)

Running title: Phylogenetic analysis of *mcr-1*-carrying *E. coli* isolates

Supplementary Figure 1

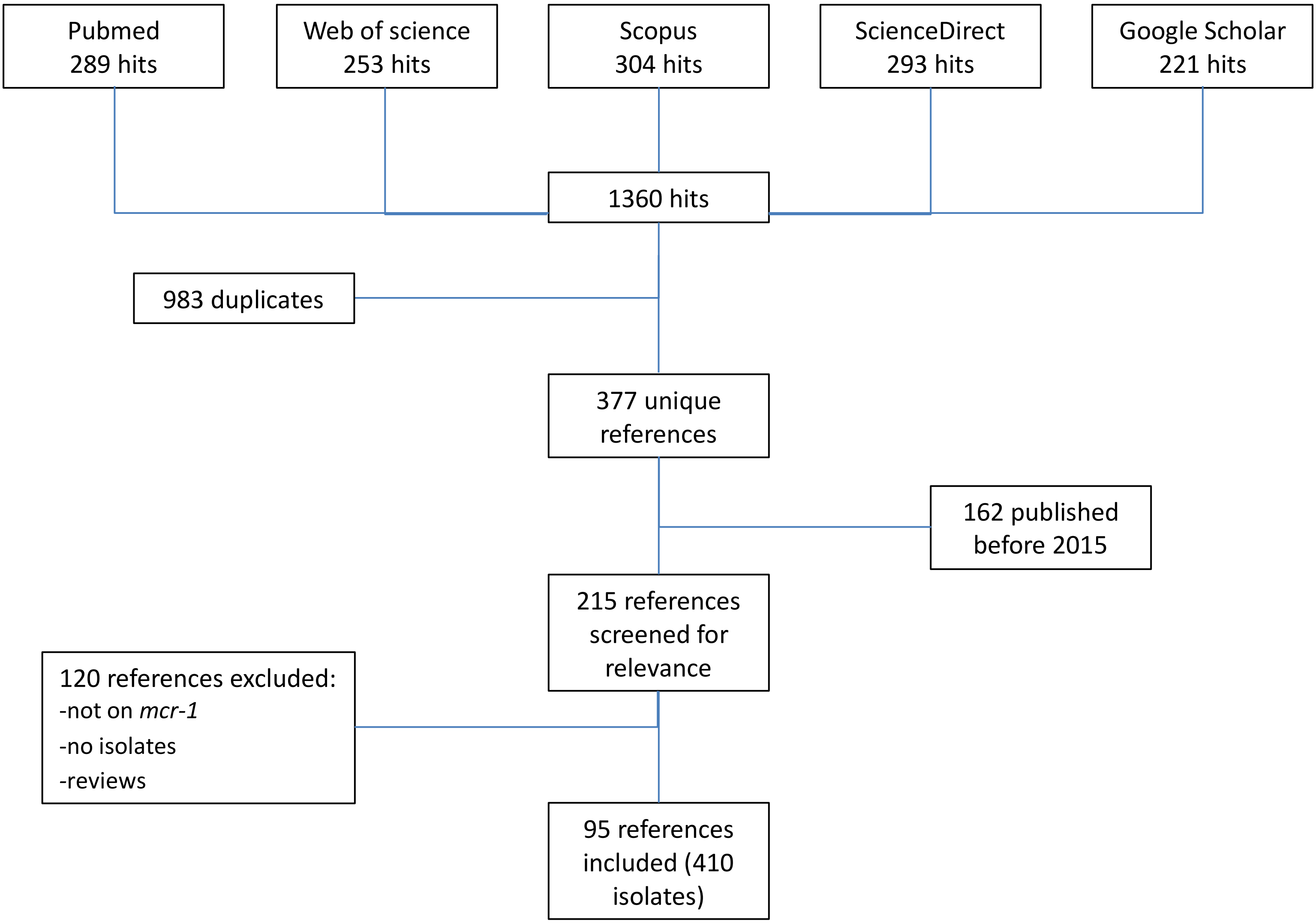

Supplementary Figure 1: Flow chart of the systematic literature review according to the PRISMA guidelines.

Systematic literature review

Relevant papers that had published on *mcr-1* and *mcr-2* were identified in Pubmed, Web of Science, Scopus, ScienceDirect and Google Scholar using the query ‘mcr-1 OR mcr1 OR mcr-2 OR mcr2 OR (mcr AND colistin)’. The database was accessed on 4 January 2017.

Criteria for considering studies for this study

Eligible for inclusion were all studies publishing:

- a multilocus sequence typing (MLST) profile (sequence type; ST) of at least one *mcr-1* positive *E. coli* isolate, or
- a whole genome sequence (WGS) of any species. For *E. coli* species the WGS was used for both phylogeny and plasmid analysis. For other species the WGS was used only for plasmid analysis, or
- the incompatibility group of a plasmid carrying a *mcr-1* or *mcr-2* gene.

All studies which did not satisfy these criteria were excluded. Studies presenting data on the same isolate were included only once.

Selection of studies

Two authors (SM and JvH) independently assessed the titles and abstracts of studies identified in terms of their relevance. Full versions of articles were obtained if the initial assessment of these met the inclusion criteria.

A flow chart of the search strategy is presented in Supplementary Figure 1. A complete list of included isolates and plasmids and their references can be found in Supplementary Table 1.

The number of entries (WGS, MLST profile, plasmid sequence or plasmid type) per included study ranged from 1 to 42 (average = 4.3; median = 2).

Search strategies

Pubmed, 289 hits

mcr1[All Fields] OR

mcr-1[All Fields] OR

mcr2[All Fields] OR

mcr-2[All Fields]) OR

("mcr"[All Fields] AND ("colistin"[MeSH Terms] OR "colistin"[All Fields])

Web of Science [v.5.23], 253 hits

TOPIC: (mcr1 OR mcr-1 OR mcr2 OR mcr-2 OR (colistin AND mcr))

Timespan: All years. Indexes: SCI-EXPANDED, SSCI, A&HCI, ESCI.

Scopus, 304 hits

(TITLE-ABS-KEY (mcr1)) OR (TITLE-ABS-KEY (mcr-1)) OR (TITLE-ABS-KEY (mcr2)) OR (TITLE-ABS-KEY (mcr-2)) OR (TITLE-ABS-KEY (colistin AND mcr))

Science direct, 293 hits

"mcr1" OR "mcr-1" OR "mcr2" OR "mcr-2" OR (colistin AND mcr) AND LIMIT-TO(yearnav, "2017,2016,2015").

Google scolar, 221 hits

mcr1 OR mcr-1 OR mcr2 OR mcr-2 OR (colistin AND mcr)

Supplementary Figure 2

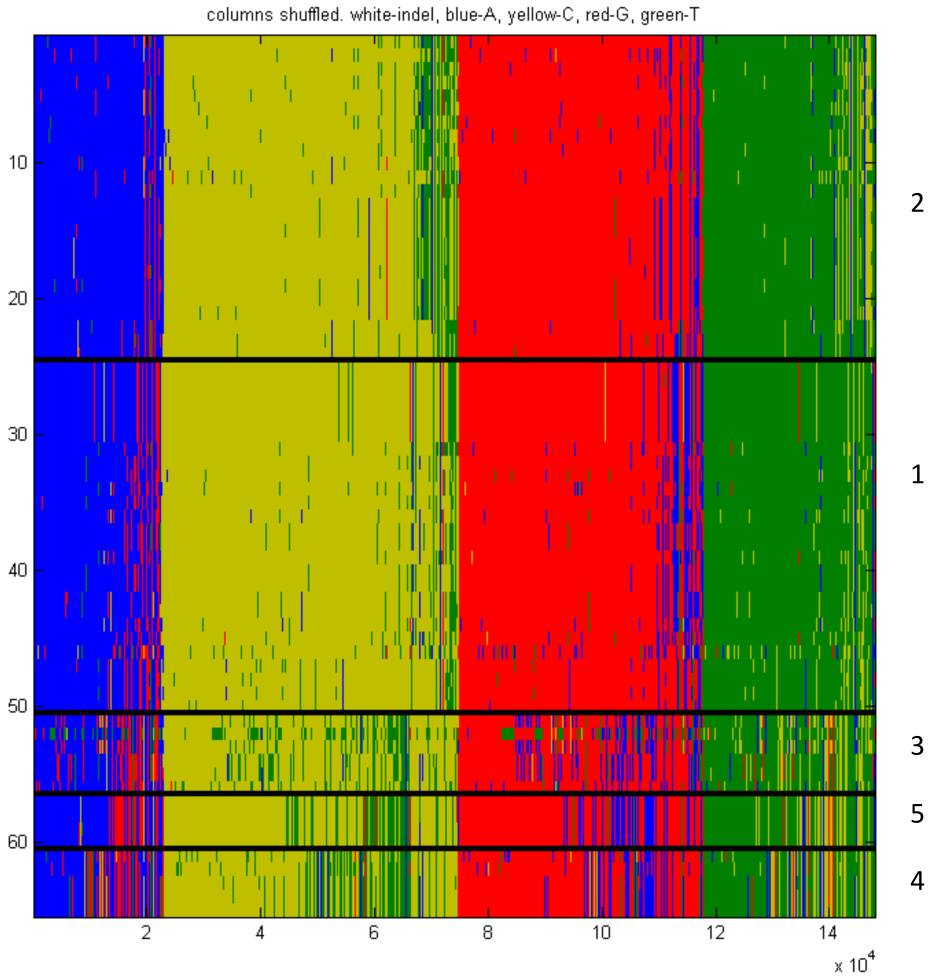

Supplementary Figure 2: Output alignment of the BAPS clustering. A shuffled alignment of the core genome SNPs is divided by black bars to indicate five BAPS groups. Each row represents an isolate and the colors in every column represent a different nucleotide. On the left-side X-axis the number of nucleotides is indicated. Clusters were numbered from 1 to 5 according to the number of isolates they contain and cluster numbers are indicated on the right-side X-axis.

White-indel = insertion or deletion; blue-A = adenine; yellow-C = cytosine; red-G = guanine; green-T = thymine

SNP counts and clonal relationships of the isolates

SNP count was performed using kSNP v3.021 [1] using a k-mer size of 19 bp and retaining only core SNPs (SNPs present in the core genome). The calculation of the genetic distance was performed using the average size of the genomes observed (4.9 Mbp). A Hamming distance matrix was created comparing the SNP/Mbp distance between each pair of isolates in the study (Supplementary Table 3).

Among the 2080 pairs of isolates studied, 20 showed between 0 and 10 SNPs/Mbp differences and were considered closely related clones (Supplementary Table 4).

Among these, 13 pairs came from live chicken from southern Vietnam and were distributed across 5 farms (Supplementary Table 4). These results suggest regional circulation of *mcr-1* carrying clones. A pair of isolates originating from the Netherlands showed 0 SNP/Mbp difference and was isolated from the same batch of chicken meat. Another pair, originating from Malaysia, was isolated from the same chicken. These two pairs of isolates most probably represent duplicates. In only one of these isolates (EC5, from Malaysia) could the plasmid incompatibility group be identified (IncI2). Thus removing the pairs of isolates from the analysis did not modify the statistical outcome of the plasmid geographical distribution. Two closely related isolates sequenced for the present study (5.5 SNPs/Mbp difference) were recovered from travellers to China and Tunisia respectively. However, they carried the *mcr-1* gene on different plasmids (IncI2 and IncHI2 respectively). These 2 isolates also differed by less than 10 SNPs/Mbp with an isolate recovered from a patient in Denmark that carried an IncI2 plasmid. An isolate recovered from a traveller to South-America, formed a pair with an isolate from a patient in Malaysia. The plasmid type could not be identified for these 2 isolates. Finally a pair of isolates belonging to ST131 and showing 5.5 SNPs/Mbp difference between them were recovered from a patient in Spain and a chicken in Germany. These two isolates carried the *mcr-1* gene on two different plasmid types, IncX4 and IncHI2 respectively.

These findings implicate that, despite the spread of certain *E. coli* clones to different continents, the *mcr-1*-carrying plasmids are acquired separately in most cases.

WGS – quality check

A total of 68 *mcr-1*-carrying *E. coli* genomes were analysed, including those 6 from travellers that were sequenced for the purpose of the present study. WGS datasets of 3 previously published isolates did not pass quality check and were discarded from the analysis. Two of these isolates (BB1290 [11.5 Mbp] and PO155 [8.5 Mbp]) had an unusually large genome. When tested using KmerFinder 2.0, the WGS fastq files of BB1290 and PO155 exhibited k-mer scores suggesting the presence of sequences from a different species in addition to the expected *E. coli*: *Enterobacter cloacae* and *Salmonella enterica* respectively. For isolate STEC-CQ10, only the sequences of the identified genes were available rather than the complete genome sequence or scaffolds. This resulted in a very fragmented genome (n = 6903 contigs), lack of intergenic regions and ultimately a serious decrease in quality of the population core genome determination.

N50 values, a statistic that defines assembly quality, for these isolates were 1096 for STEQ-CQ10; 14079 for PO155 and 31281 for BB1290. These values were much lower than values observed for the other genomes of the collection (N50 > 100000).

All these results taken together resulted in the exclusion of the WGS of STEQ-CQ10, BB1290 and PO155 from the phylogenetic analysis.

Supplementary Figure 3

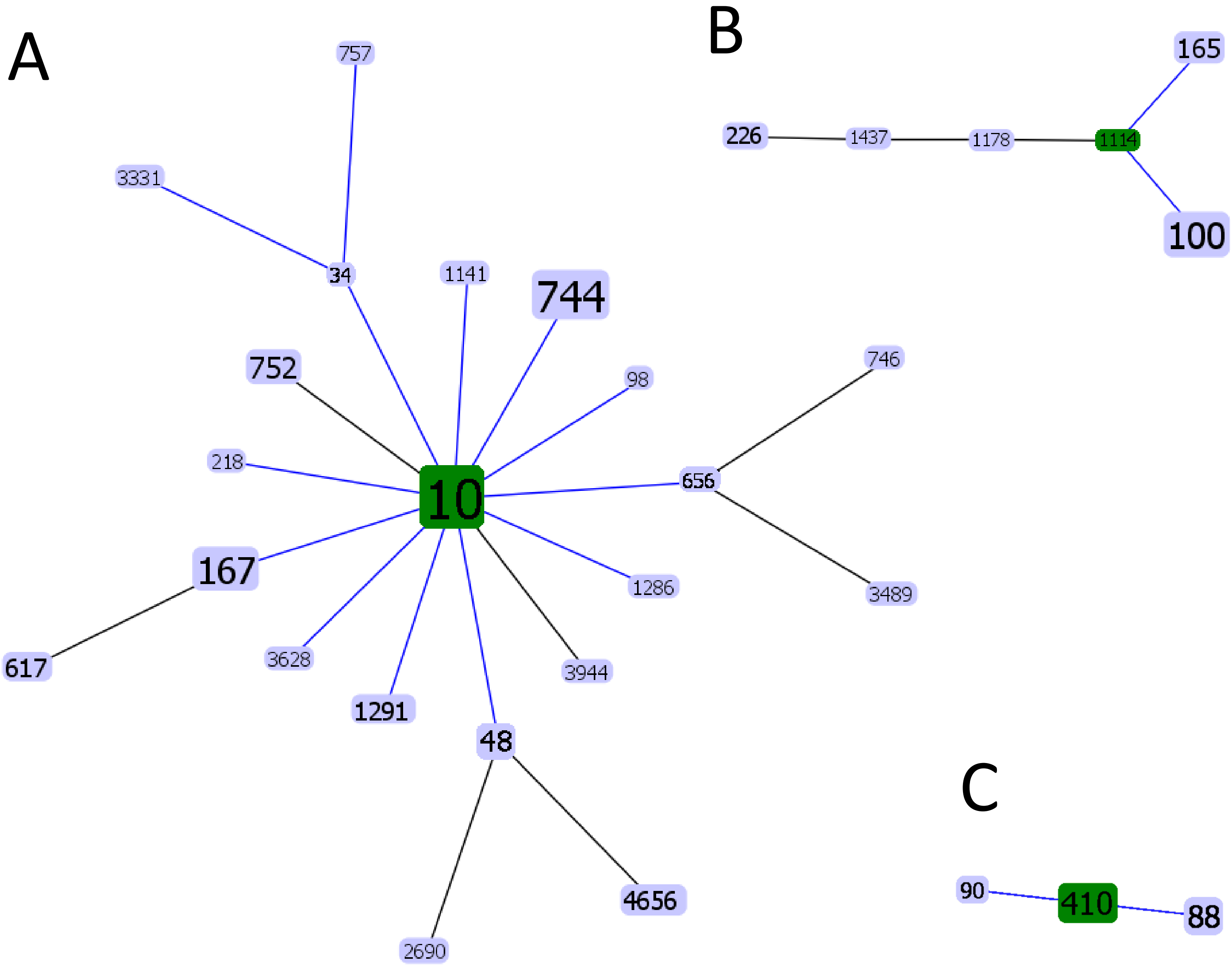

Supplementary Figure 3: representation of the eBURST analysis showing the MLST clonal relationships of the 3 largest clusters containing 120 of the 312 included *mcr-1*-carrying *E. coli* isolates. Each rectangle represents a distinct sequence type (ST), with the number inside the rectangle referring to the ST's nomenclature according to the University of Warwick MLST database. The predicted founder ST of each group is indicated in green. Black lines indicate links drawn without recourse to tiebreak rules by goeBURST [2]; Blue lines indicate links drawn using tiebreak rule. The size of the rectangles that indicate the STs are proportional to the number of isolates they contain.

A: eBURST cluster 1 (n = 86 isolates).

B: eBURST cluster 2 (n = 20 isolates).

C: eBURST cluster 3 (n = 14 isolates).

Supplementary Figure 4

A

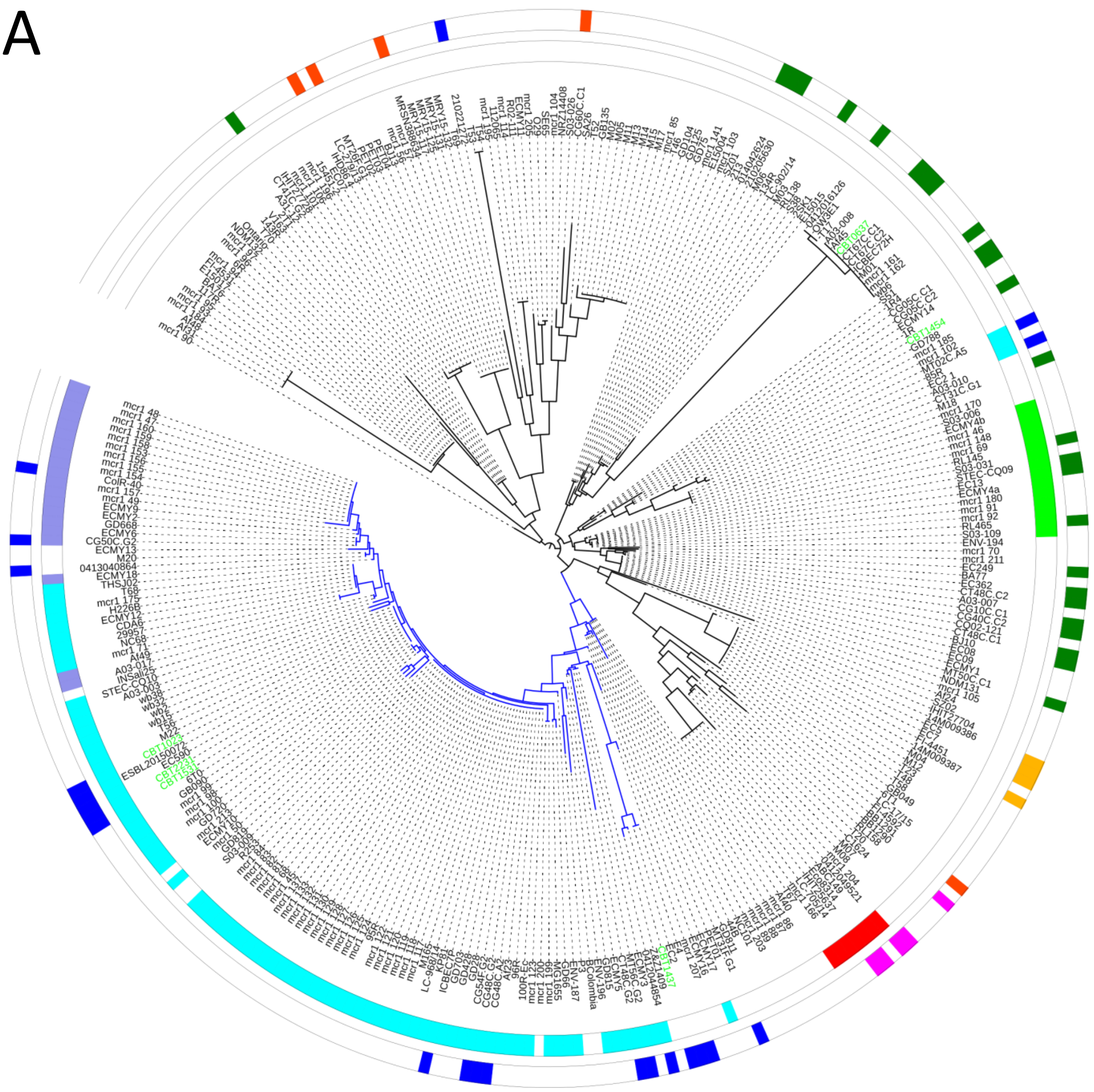

eburst_clusters*	Color Key
1 (ST10)	
2 (ST1114)	
3 (ST410)	
ST131	

BAPS cluster	Color key
1	
2	
3	
4	
5	

Tree scale: 0.001

Supplementary Figure 4

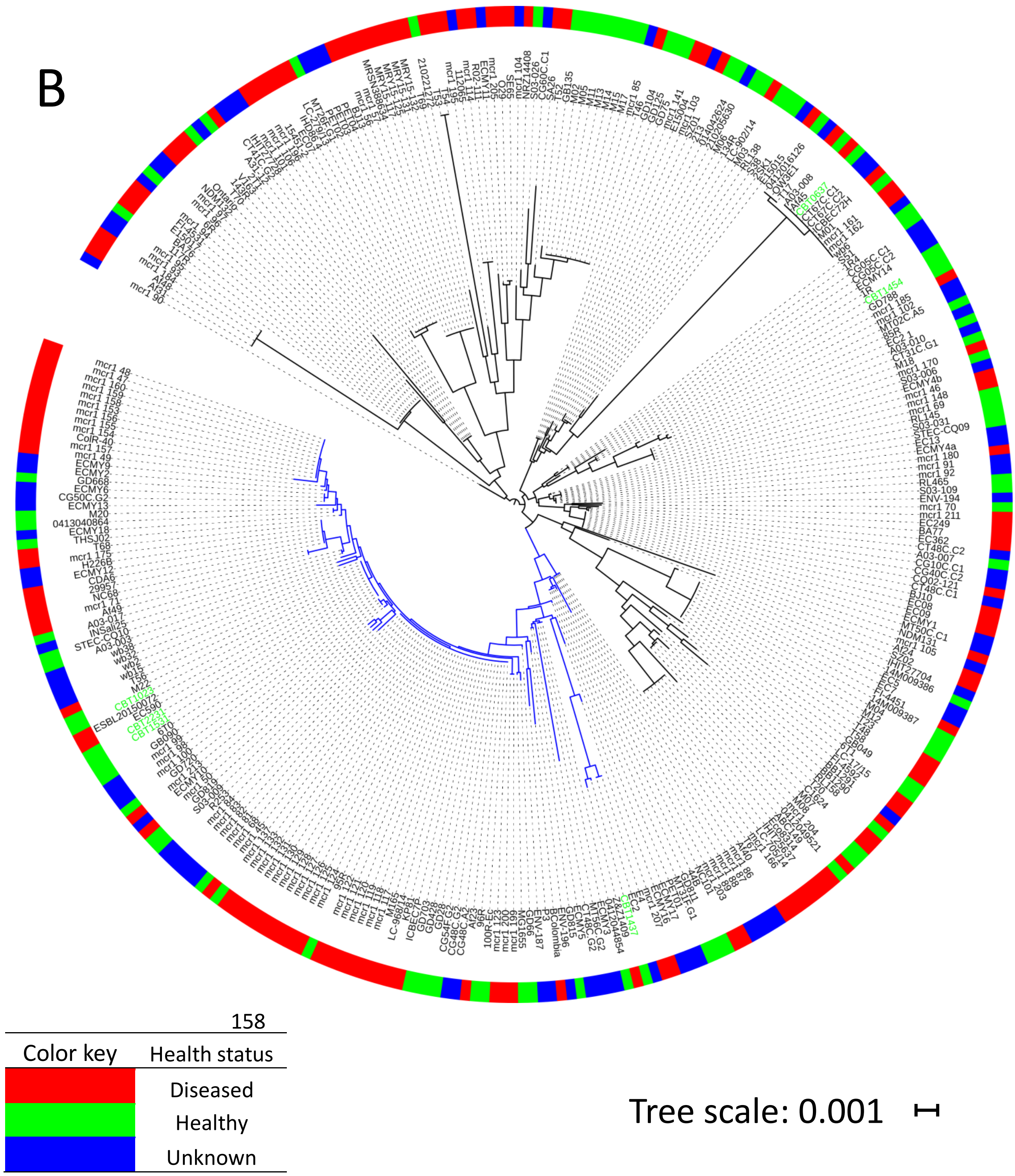

Supplementary Figure 4: Phylogeny of 312 *mcr-1*-carrying *E. coli* isolates. Maximum-likelihood trees based on concatenated MLST gene sequences, mid-point rooted.

The 6 travellers' isolates that were sequenced for this study are highlighted in green. The branch of the main clade containing most isolates from ST10 is coloured in blue. Tree scale in number of substitutions per site.

A: Colours of the inner circle indicate in which eBURST cluster (SLVs and DLVs clusters) the isolates belonged after MLST analysis; no colour indicates that the isolates were not part of one of the 3 indicated clusters. ST131 is not part of any eBURST cluster and is indicated separately. Colours of the outer circle indicate in which BAPS cluster the isolates belonged after whole genome sequence (WGS) analysis. No colour indicates that no WGS was available.

*The predicted founder ST of each cluster is indicated in brackets.

B: Colours of the circle indicate the health status of the host from which the isolate was recovered, if available.

MLST analysis

Four different STs consisting of more than 2 isolates that were possibly clonally related were found in a single region: ST100 (10 isolates) and ST752 (3 isolates) in Europe; ST156 (12 isolates) and ST165 (3 isolates) in Asia. However all 3 isolates from ST752 were reported in a single study [3], as were 8 out of 10 isolates from ST100 [4].

Supplementary Figure 5

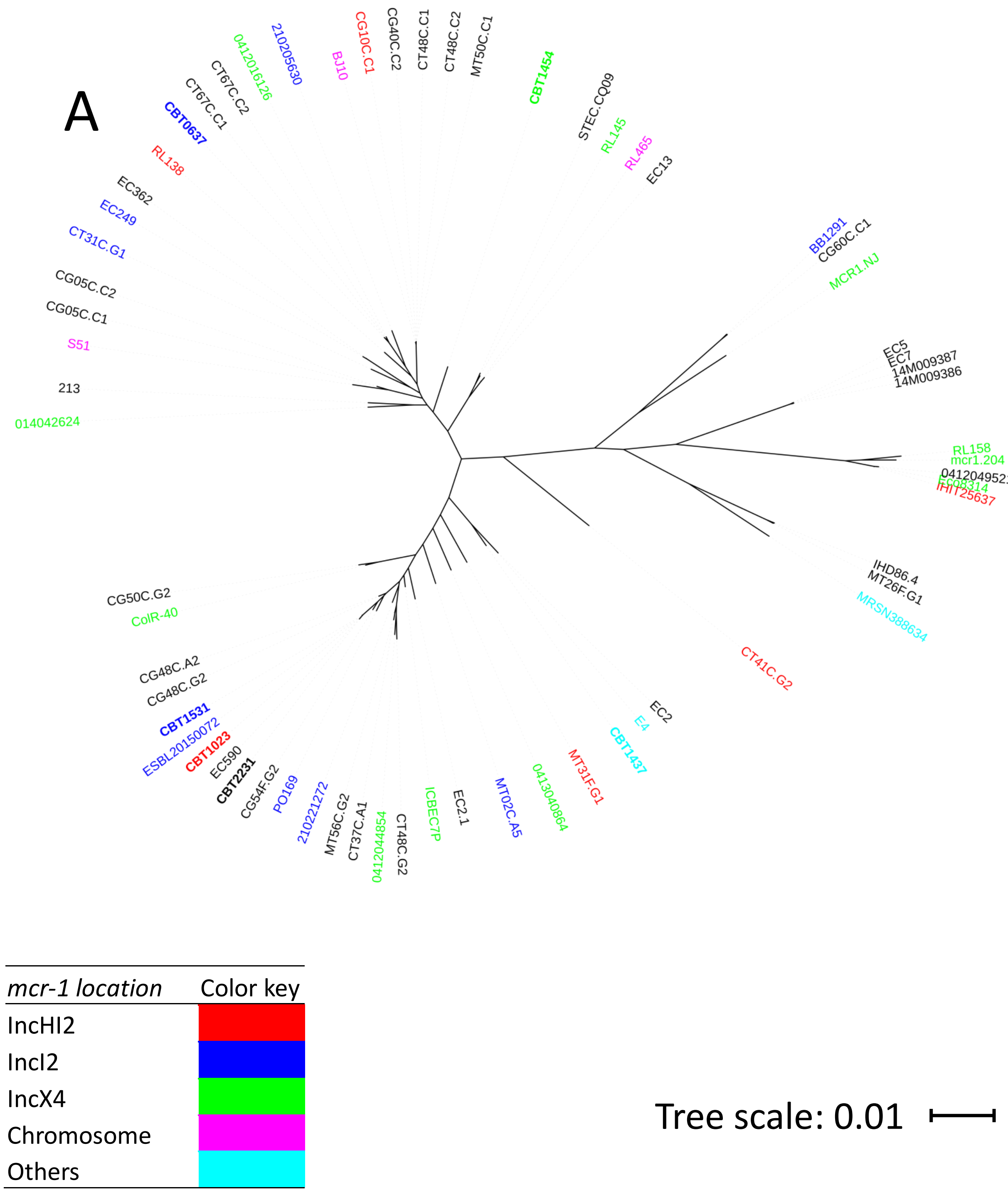

Supplementary Figure 5

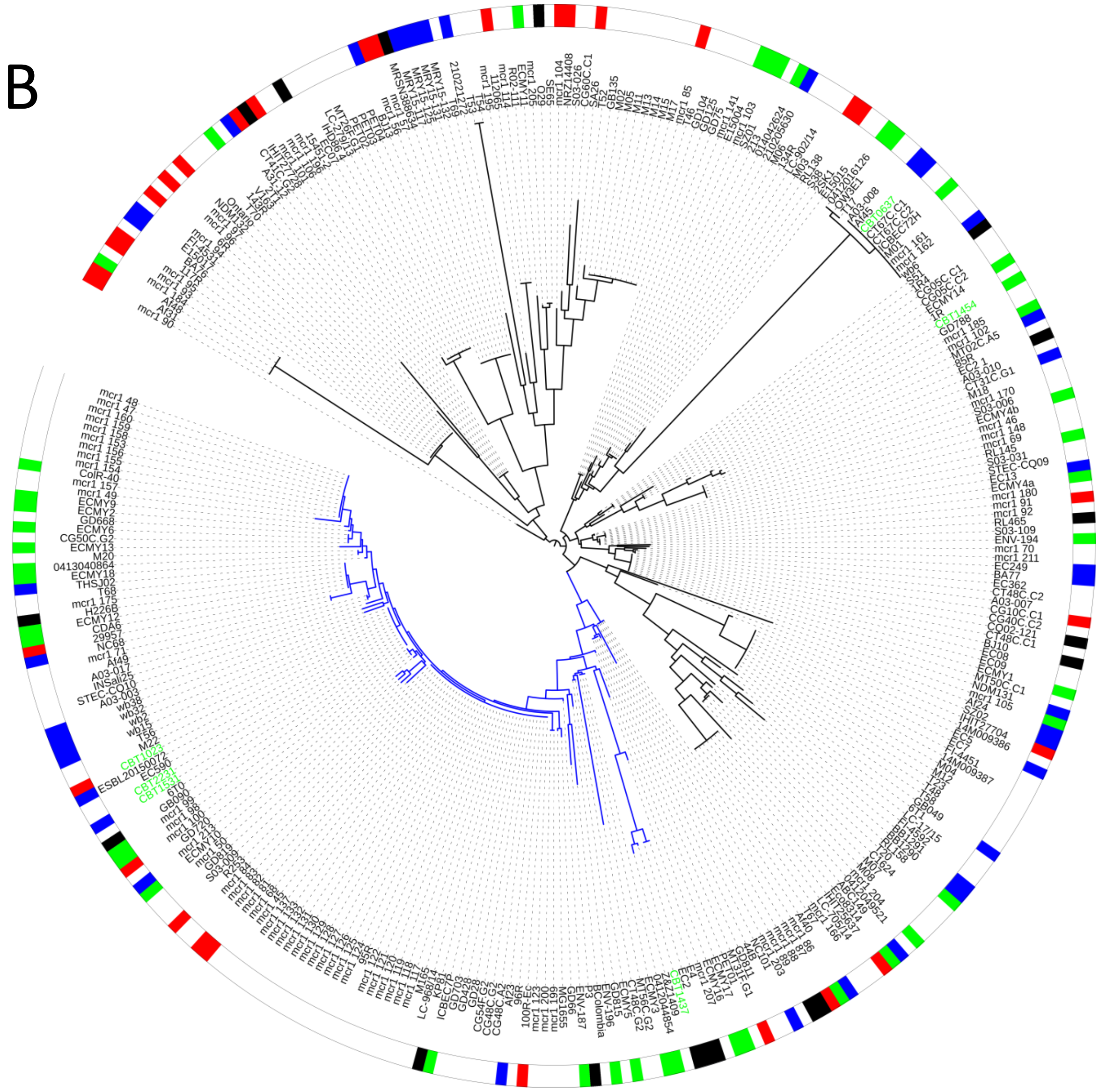

mcr-1 location	Colour key
IncHI2	
IncI2	
IncX4	
Chromosome	
Other	
Unknown	

Supplementary Figure 5: Association between location of the *mcr-1* gene and WGS and MLST phylogeny of population of studied *mcr-1*-carrying *E. coli* isolates.

Only plasmids IncHI2, IncI2 and IncX4 are indicated by separate colours, all other plasmid types are indicated as “other”. Trees scales in number of substitutions per site.

A: Maximum-likelihood tree based on concatenated core genome sequences of the 65 *mcr-1*-carrying *E. coli* isolates. Isolate names are coloured according to the location of the *mcr-1* gene, either on an identified plasmid, chromosome of the isolate or unknown location. The 6 travellers' isolates that were sequenced for this study are highlighted in bold.

B: Maximum-likelihood tree based on concatenated MLST gene sequences, mid-point rooted.

Colours indicate the location of the *mcr-1* gene. The branch of the main clade containing most isolates from ST10 is coloured in blue. The 6 travellers' isolates that were sequenced for this study are highlighted in green.

Antimicrobial resistance genes

Using the ResFinder tool, a total of 65 different unique acquired antimicrobial resistance (AMR) genes were identified in the *E. coli* WGS dataset. Many additional chromosomally encoded AMR genes were found using the CARD database. The most prevalent acquired gene (after *mcr-1*) was *tetA*, found in 52/65 (80%) of isolates followed by *sul2* (39/65; 60%) and the beta-lactamase gene TEM-1b in 38 isolates (58%). One isolate only carried just one additional acquired gene, the AmpC beta-lactamase gene *blaCMY-2*, and one isolate carried no other acquired AMR gene apart from *mcr-1*. In addition to *mcr-1*, each of the 65 isolates carried 4 or 5 genes of the *pmr* family (A, B, C, E and F variants). These genes are involved in the resistance to polymyxin, but are present in all *E. coli* strains [5]. Genetic alignments showed SNPs differences between the *pmrA* and *B* genes found in this study and both the sensitive and resistant templates previously described [5], thus preventing from drawing any conclusion relative to the involvement of these genes in the colistin resistance mechanisms. Carbapenemase encoding genes were found in the genomes of 4 isolates; NDM-5 (in isolates BJ10 and MCR1.NJ from China and USA respectively) and KPC-2 (EC362 and EC249, both from Singapore). 29/65 isolates (44.6%) carried plasmid-encoded CTX-M ESBL genes with CTX-M-55 being most prevalent in 18/29 (62.1%) of these isolates. Ten (15.4%) isolates were found to carry plasmid mediated *Qnr* quinolone resistance genes, 9 isolates carried *qnrS1* and one both *qnrS1* and *qnrS2*. In addition, 36 (55.4%) isolates displayed chromosomal mutations in both *parC* and *gyrA* genes associated with resistance to (fluoro)quinolones [6]. 34 (52.3%) isolates carried the *strA* and *strB* streptomycin resistance genes. The florfenicol resistance gene *floR* was present in 32 (49.2%) isolates; in 22 of 45 isolates from animals (48.9%) and 10 of 19 from humans (52.6%). The *baeR* and *baeS* genes are regulators of resistance mechanisms to novobiocin [7,8] and were both found in 64 (98.5%) and 65 (100%) isolates, respectively.

Supplementary data references

1. Gardner, S. N., Slezak, T. & Hall, B. G. kSNP3.0: SNP detection and phylogenetic analysis of genomes without genome alignment or reference genome. *Bioinformatics* **31,** 2877–2878 (2015).

2. Francisco, A. P., Bugalho, M., Ramirez, M. & Carriço, J. a. Global optimal eBURST analysis of multilocus typing data using a graphic matroid approach. *BMC Bioinformatics* **10,** 152 (2009).

3. Veldman, K. *et al.* Location of colistin resistance gene mcr-1 in Enterobacteriaceae from livestock and meat. *J. Antimicrob. Chemother.* **71,** 2340–2342 (2016).

4. El Garch, F. *et al.* mcr-1 is borne by highly diverse Escherichia coli isolates since 2004 in food-producing animals in Europe. *Clin. Microbiol. Infect.* **23,** 51e1-51e4 (2017).

5. Quesada, A. *et al.* Polymorphism of genes encoding PmrAB in colistin-resistant strains of Escherichia coli and Salmonella enterica isolated from poultry and swine. *J. Antimicrob. Chemother.* **70,** 71–74 (2015).

6. Bagel, S., H??llen, V., Wiedemann, B. & Heisig, P. Impact of gyrA and parC mutations on quinolone resistance, doubling time, and supercoiling degree of Escherichia coli. *Antimicrob. Agents Chemother.* **43,** 868–875 (1999).

7. Lilic, M., Jovanovic, M., Jovanovic, G. & Savic, D. J. Identification of the CysB-regulated gene, hslJ, related to the Escherichia coli novobiocin resistance phenotype. *FEMS Microbiol. Lett.* **224,** 239–246 (2003).

8. Baranova, N. & Nikaido, H. The BaeSR Two-Component Regulatory System Activates Transcription of the yegMNOB (mdtABCD) Transporter Gene Cluster in Escherichia coli and Increases Its Resistance to Novobiocin and Deoxycholate. *J. Bacteriol.* **184,** 4168–4176 (2002).
